# Loss of ATF2 Function Leads to Cranial Motoneuron Degeneration during Embryonic Mouse Development

**DOI:** 10.1371/journal.pone.0019090

**Published:** 2011-04-21

**Authors:** Julien Ackermann, Garry Ashton, Steve Lyons, Dominic James, Jean-Pierre Hornung, Nic Jones, Wolfgang Breitwieser

**Affiliations:** 1 Cell Regulation Department, Paterson Institute for Cancer Research, University of Manchester, Manchester, United Kingdom; 2 Institut de Biologie Cellulaire et de Morphologie, Lausanne, Switzerland; Universidade Federal do Rio de Janeiro, Brazil

## Abstract

The AP-1 family transcription factor ATF2 is essential for development and tissue maintenance in mammals. In particular, ATF2 is highly expressed and activated in the brain and previous studies using mouse knockouts have confirmed its requirement in the cerebellum as well as in vestibular sense organs. Here we present the analysis of the requirement for ATF2 in CNS development in mouse embryos, specifically in the brainstem. We discovered that neuron-specific inactivation of ATF2 leads to significant loss of motoneurons of the hypoglossal, abducens and facial nuclei. While the generation of ATF2 mutant motoneurons appears normal during early development, they undergo caspase-dependent and independent cell death during later embryonic and foetal stages. The loss of these motoneurons correlates with increased levels of stress activated MAP kinases, JNK and p38, as well as aberrant accumulation of phosphorylated neurofilament proteins, NF-H and NF-M, known substrates for these kinases. This, together with other neuropathological phenotypes, including aberrant vacuolisation and lipid accumulation, indicates that deficiency in ATF2 leads to neurodegeneration of subsets of somatic and visceral motoneurons of the brainstem. It also confirms that ATF2 has a critical role in limiting the activities of stress kinases JNK and p38 which are potent inducers of cell death in the CNS.

## Introduction

ATF2 belongs to the leucine-zipper domain-containing CREB/ATF transcription factor family. It binds DNA as a homodimer on calcium/cAMP response element (CRE) sequences or as a heterodimer with structurally related AP-1 proteins, such as c-Jun, to control the expression of a variety of target genes [1]. ATF2 is a substrate for MAP kinases, including c-Jun N-terminal kinase (JNK), p38 kinase and p44/p42 MAPK (ERK1/2) [2], [3], [4]. MAPK phosphorylation of two threonine (Thr) residues, Thr69 and Thr71, is required for transcriptional activation of ATF2 [4], [5]. One major role of ATF2 is to regulate the response of cells to stress signals and JNK- and p38-mediated phosphorylation of ATF2 is a key process for this response [6], [7], [8], [9]. ATF2 has also been shown to be phosphorylated by ATM kinase in response to DNA damage [10].

ATF2 mutant mice generated by different gene targeting approaches have demonstrated the importance of ATF2 for tissue development and integrity and for postnatal viability. A hypomorphic ATF2 mouse mutant uncovered a variety of developmental abnormalities, leading to defects in endochondral ossification as well as defects in the nervous system. The latter include ataxia, a reduced number of cerebellar Purkinje cells, atrophic vestibular sense organs and enlarged ventricles, demonstrating the importance of ATF2 for the coordinated development of the mammalian brain [11]. A transcriptional null ATF2 mouse mutant line is lethal at birth due to severe respiratory distress resembling the human meconium aspiration syndrome [12]. Furthermore, a knock-in mutant line in which the Thr69 and Thr71 phosphorylation sites are mutated into alanines (*Atf2^AA^*) leads to a similar phenotype and to invariable death at birth, confirming the importance of these phosphorylation sites for ATF2 activity [13].

In the nervous system, the role of ATF2 and of its binding partner c-Jun in neurodegenerative stimuli-induced cell death has been extensively studied *in vivo*. Phosphorylation of ATF2 at Thr71 was shown to occur during reperfusion following transient cerebral ischemia in rat CA1 hippocampal neurons that were irreversibly damaged [14]. In addition, expression and activation of c-Jun was shown to have a significant role in neural development as well as in a number of different neuronal pathologies [15]. Although ATF2 can regulate the expression of the *c-Jun* gene, substantial evidence suggests that ATF2 and c-Jun have different roles in neuronal cells. ATF2 is highly expressed in neurons of the adult rat nervous system except for those neuronal populations which exhibit a high basal level of c-Jun, such as the dentate gyrus, the red nucleus and some cranial and spinal cord motoneurons (i.e. hypoglossal, facial, oculomotor and sciatic nuclei) [16]. Following transection of peripheral or central nerve fibres, such as the optic nerve or the vagal and facial nerve fibres, ATF2 levels rapidly decreased in the axotomised neurons during the period in which c-Jun expression was rapidly increased [16]. ATF2 expression is also rapidly suppressed following ischemia and after mechanical injury during the process of degeneration [17]. These results indicate that the interplay between ATF2 and c-Jun activities is uncoupled in neuronal cells. To understand in more detail the role of ATF2 in neuron survival, we generated a neural cell-specific, conditional mouse mutant for ATF2. We show that specific deletion of ATF2 in neurons leads to death after birth with similar phenotypic appearances as the knockout germ line mutation. In these mutant mice we find severe developmental defects in essential motoneurons of the hindbrain with impact on respiratory regulation, an observation which underlines the phenotypic abnormalities seen at birth.

## Results

### Lack of functional ATF2 impairs proper development of specific regions in the hindbrain

To produce a neuronal cell-specific ATF2 deletion, we crossed *nestin-Cre* mice with mice expressing a floxed allele of *Atf2* (*Atf2^f/f^*, see Materials and Methods). *Nestin-Cre* induced recombination has been shown to be efficient and tissue specific from early stages of CNS development [18]. The crosses led to the effective deletion of the DNA binding domain of ATF2 as normal ATF2 protein was no longer detected in E18.5 brain of *Atf2^f/f^;nestin-Cre* mice (*Atf2^Δneuron^*) (Figure S1A). Surprisingly, no adult *Atf2^Δneuron^* animals emerged, suggesting that neuronal loss of ATF2 is lethal. We then analysed when the lethality occurred and found that *Atf2^Δneuron^* embryos were born at the expected frequency. However, the mutant newborns were cyanotic and in respiratory distress, and invariably died shortly after birth (Table 1). This phenotype was very reminiscent of a previous finding of early postnatal death of *Atf2^−/−^* mice [13]. Our findings suggest that the underlying defect for this lethality was situated within the CNS while the early postnatal death observed by Maekawa et al. (1999) was attributed to defects in placental functions leading to stress-induced meconium aspiration during the birth process. To clarify this, we generated an additional conditional knockout using *meox2-Cre* as the driver [19] resulting in ATF2 deleted in the epiblast and subsequently in all embryonic tissues except for the placenta *Atf2^Δepiblast^*). Like *Atf2^Δneuron^* and *Atf2^−/−^* mice, the *Atf2^Δepiblast^* mutation also led to early postnatal death suggesting that the loss of ATF2 dependent phenotypes cannot be attributed to placental defects but rather to embryonic and primarily neuronal defects.

**Table 1:** List of germline and tissue specific conditional ATF2 deletions.

Genotype	Mutation	Phenotype
*Atf2^−/−^*	Germline deletion	100% lethality at birth
*Atf2^AA^*	T51/T53 mutation	Same as *Atf2^−/−^*
*Atf2^Δneuron^*	*Atf2^f/f^;nestin-Cre*	Same as *Atf2^−/−^*
*Atf2^Δepiblast^*	*Atf2^f/f^;meox2-Cre*	Same as *Atf2^−/−^*

Atf2*^−^* ^/*−*^ mutant mice were obtained as a result of Atf2^+/*−*^ x Atf2^+/*−*^ crosses. Atf2^AA^ were the result of Atf2^+/AA^ x Atf2^+/AA^ crosses. Atf2^Δneuron^ and Atf2^Δepiblast^ resulted from Atf2^f/f^ x Atf2^f/+^;nestin-Cre and Atf2^f/f^ x Atf2^f/+^;meox2-Cre crosses, respectively. All animals were on a C57/Bl6 strain background. A minimum of 10 offspring litters were analysed for each knockout combination.

**Figure 1:**
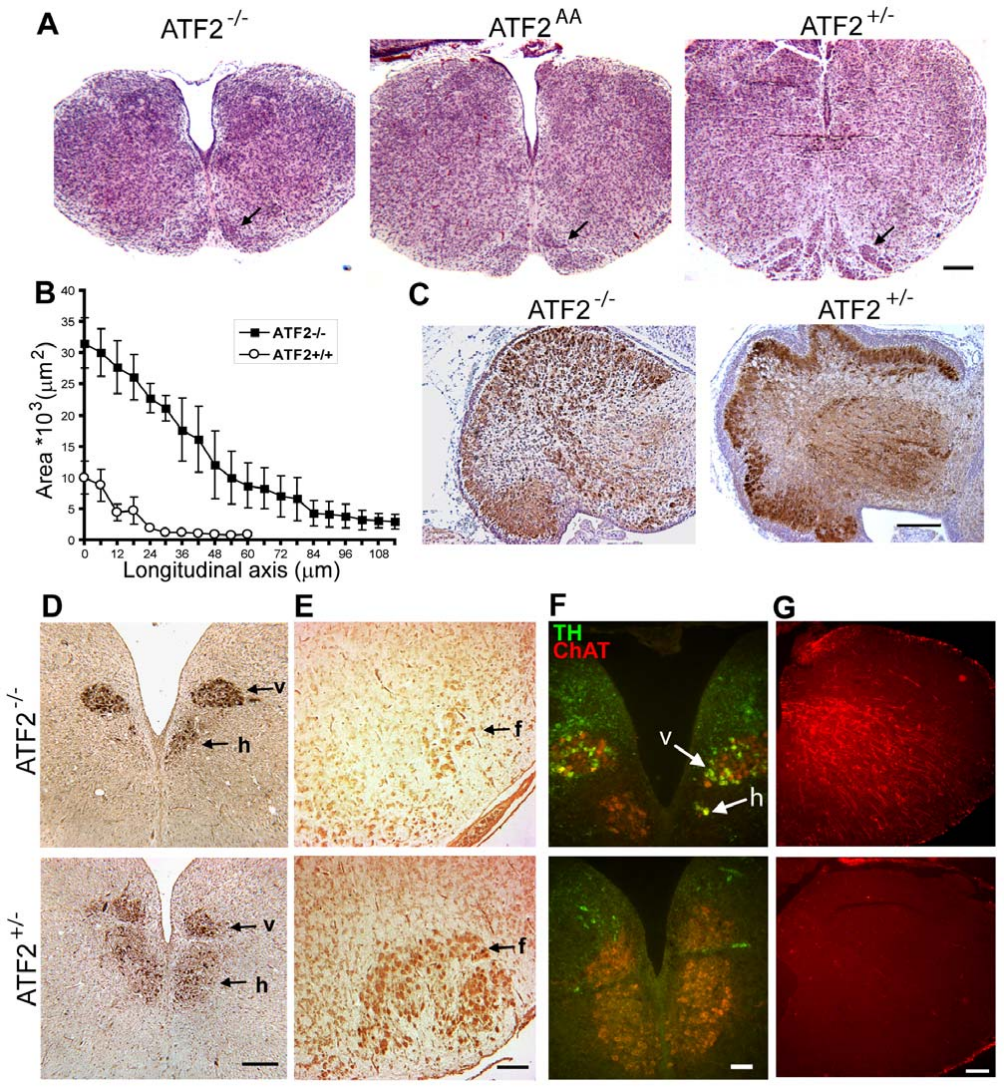
Histological abnormalities in E18.5 ATF2 mutant cerebellum and brainstem. (A) Hematoxylin and eosin (H&E) stained transversal sections of brainstem at the level of the inferior olive. *Atf2^−/−^* and *Atf2^AA^* brainstems are significantly smaller and have an enlarged central canal compared to *Atf2^+/−^*. The inferior olive (arrow) is severely underdeveloped in mutant embryos. Bar: 250 µm. (B) Area plot of the central canal (mean ± SEM of 3 brainstems analysed per genotype), measured along the antero-posterior axis of serial transversal sections from the obex (0 µm on the x-axis) posteriorly to the caudal end of the medulla, shows a significant enlargement of the canal in *Atf2^−/−^* embryos. (C) Horseradish peroxidase (HRP) immunostaining of calbindin in sagittal sections of the cerebellum. *Atf2^−/−^* cerebellum lacks the foliation and the tripartite layering seen in *Atf2^+/−^* cerebellum. Bar: 250 µm. (D) HRP immunostaining of choline acetyltransferase (ChAT) in transversal sections of *Atf2^−/−^* and *Atf2^+/−^* posterior medulla. Number of hypoglossal motoneurons (h) is bilaterally decreased in *Atf2^−/−^* embryos while number of dorsal vagal motoneurons (v) appears normal. This was seen at several levels along the longitudinal axis. Bar: 200 µm. (E) HRP immunostaining of Islet-1 (Isl-1) in transversal sections of *Atf2^−/−^* and *Atf2^+/−^* anterior medulla. A severe reduction in the number of motoneurons is found in the *Atf2^−/−^* facial nucleus (f). Bar: 100 µm. (F) Double immunofluorescence staining of TH (green) and ChAT (red) shows aberrant expression of TH in hypoglossal (h) and dorsal vagal (v) motoneurons in *Atf2^−/−^* brains. Bar: 50 µm. (G) GFAP immunostaining (red) reveals aberrant expression of GFAP in the mantle zone of *Atf2^−/−^* brainstem. Bar: 50 µm.

We then compared the phenotypic appearance of brains from *Atf2^+/+^* with *Atf2^−/−^*, phosphorylation deficient *Atf2^AA^* [13], and *Atf2^Δneuron^* E18.5 embryos. Overall, whole brain weights appeared similar between the different genotypes (data not shown). In contrast, cerebelli of *Atf2^−/−^*, *Atf2^AA^* and *Atf2^Δneuron^* embryos were smaller and lacked the typical foliation and tripartite layering seen in wild-type or heterozygous embryos (Figure 1C and Figure S1B and data not shown). This phenotype may likely develop into the cerebellum defect that has been described in adult hypomorphic ATF2 knockouts that were viable after birth [11]. We then examined the brainstem of control and mutant embryos. E18.5 brainstems of wild-type and heterozygous embryos were indistinguishable in all experiments that were done, so we considered ATF2 heterozygous as wild-types. The brainstem of both *Atf2^−/−^* and *Atf2^AA^* E18.5 embryos appeared significantly smaller while the neural tube, from the obex to the caudal end of the medulla, was enlarged compared to control littermates (Figure 1A and B). We also noticed that in the mutants, neurons in the inferior olive were diffusely distributed and did not form the typical crescent shape seen in heterozygous embryos (Figure 1A, arrows). Strikingly, in *Atf2^−/−^* brains, hypoglossal and facial motoneurons stained with the motoneuron-specific markers choline acetyltransferase (ChAT), and Islet-1 (Isl-1), were partly missing (Figure 1D and E) and abducens motoneurons were completely absent (see below). In contrast, and in accordance with a previous publication [20], abnormal expression of tyrosine hydroxylase (TH) was observed at different antero-posterior levels of the midbrain and hindbrain (data not shown). TH and ChAT double immunofluorescence staining revealed aberrant expression of TH in some, otherwise cholinergic, *Atf2^−/−^* hypoglossal and dorsal vagal motoneurons (Figure 1F). Furthermore, we found increased expression of the glial cell-specific marker glial fibrillary acid protein (GFAP) in the mantle zone of *Atf2^−/−^* brainstems indicative of astrogliosis (Figure 1G).

**Figure 2:**
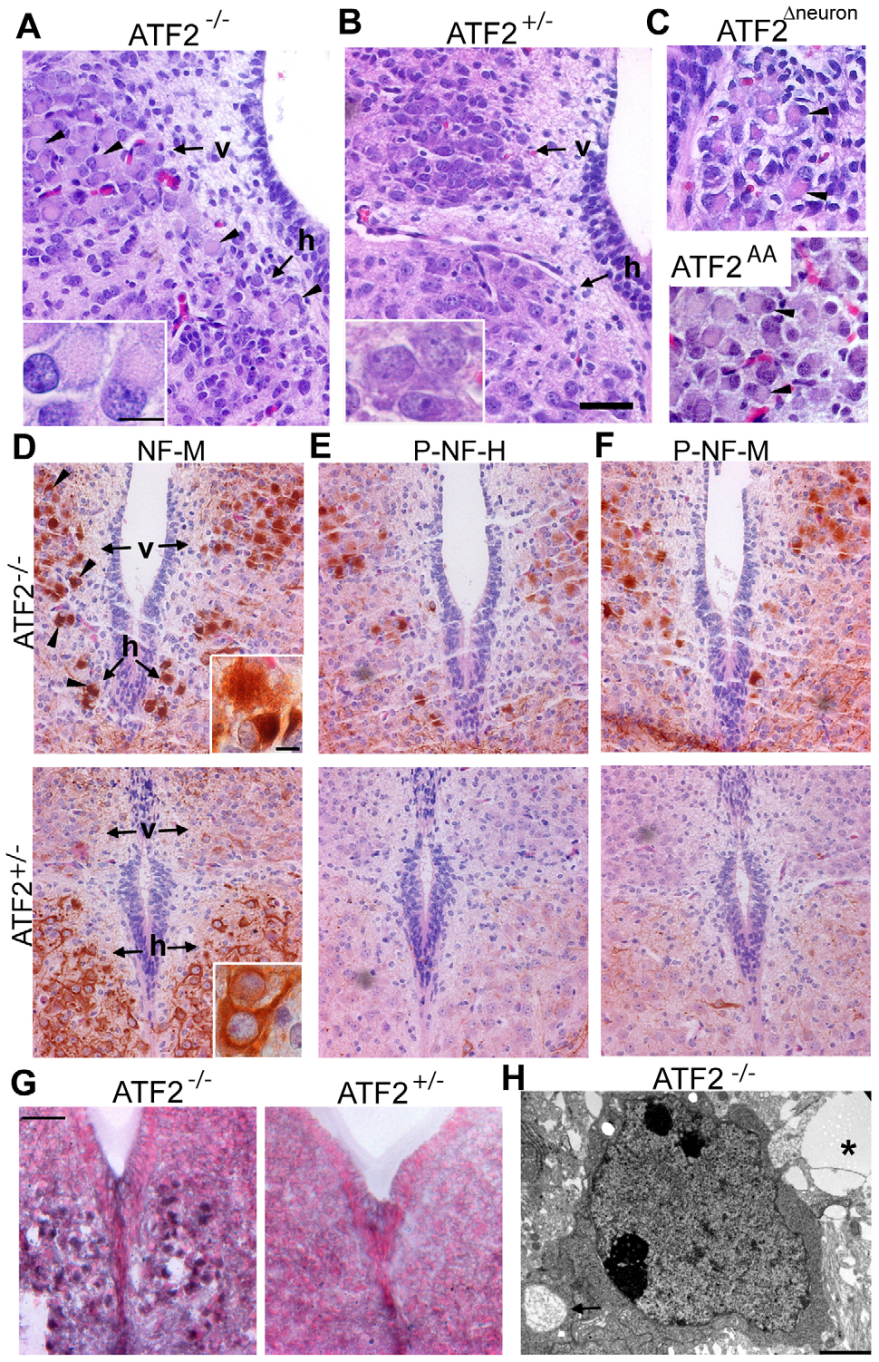
Neuropathological lesions in E18.5 ATF2 mutant motoneurons. (A, B) H&E stained transversal sections of *Atf2^−/−^* and *Atf2^+/−^* posterior medulla. *Atf2^−/−^* hypoglossal (h) and dorsal vagal (v) motoneurons show ballooned perikarya with eccentrically positioned nuclei (arrowheads). Insets: magnification of dorsal vagal motoneurons. (C) H&E staining of *Atf2^Δneuron^* hypoglossal and *Atf2^AA^* vagal motoneurons showing the same pathological lesions (arrowheads). (D–F) HRP immunostaining with antibodies against neurofilament M (NF-M), phospho-neurofilament H (P-NF-H, RMO24.9) and phospho-neurofilament M (P-NF-M, RMO8) in *Atf2^−/−^* and *Atf2^+/−^* posterior medulla. Aberrant NF-M accumulation in the soma of *Atf2^−/−^* dorsal vagal and hypoglossal motoneurons is indicated by arrowheads. P-NF-H and P-NF-M are predominantly present in *Atf2^−/−^* motoneurons. Insets: magnification of NF-M stained hypoglossal motoneurons. (G) Sudan black B staining shows lipid accumulation in *Atf2^−/−^* but not in *Atf2^+/−^* hypoglossal motoneurons. Bars: A–G, 100 µm; insets, 25 µm.(H) Transmission electron microscopy photograph of *Atf2^−/−^* brainstem shows a lipid droplet near a cell (asterisk), and a cytoplasmic vacuole filled with neurofibrillary material (arrow). Bar: 2 µm.

Hypoglossal and dorsal vagal motoneurons play an important role in respiration and cardiac function [21] and their disruption could explain the perinatal respiratory defects of ATF2 mutant mice. We therefore concentrated on further analysis of embryonic motoneuron defects. Hematoxylin and eosin (H&E) stained sections of E18.5 *Atf2^−/−^* hypoglossal and dorsal vagal motoneurons displayed ballooned perikarya with eccentrically positioned nuclei (Figure 2A and B). This phenotype was reproduced in *Atf2^AA^* and *Atf2^Δneuron^* embryos (Figure 2C), indicating that the loss of the transcriptionally active form of ATF2 impairs proper development of these neurons. Immunostaining against the cytoskeletal protein neurofilament M (NF-M) demonstrated an abnormal accumulation of neurofilament in the soma of mutant motoneurons (Figure 2D). Moreover, accumulated neurofilaments were found to be hyperphosphorylated in *Atf2^−/−^* hypoglossal and dorsal vagal motoneurons as shown by staining for phospho-NF-H and phospho-NF-M (Figure 2E and F). Neurofilament hyperphosphorylation has been found in certain neurodegenerative diseases, including amyotrophic lateral sclerosis, ALS [22]. In addition, in ALS as well as in other neurodegenerative diseases, abnormal lipid metabolism has been found [23]. Staining of *Atf2^−/−^* and *Atf2^+/−^* brainstem sections with the fat-soluble dye Sudan black B revealed an abnormal accumulation of lipids in *Atf2^−/−^* hypoglossal neurons (Figure 2G). Transmission electron microscopy on *Atf2^−/−^* brainstem sections containing the hypoglossal nucleus revealed vacuoles accumulating in cell bodies and surrounding neuropil (Figure 2H). These results therefore suggest that neurodegeneration occurs in the hypoglossal and dorsal vagal motoneurons of ATF2 mutant embryos.

### Neural patterning and axonal growth is normal in *Atf2^−/−^* brainstem

**Figure 3:**
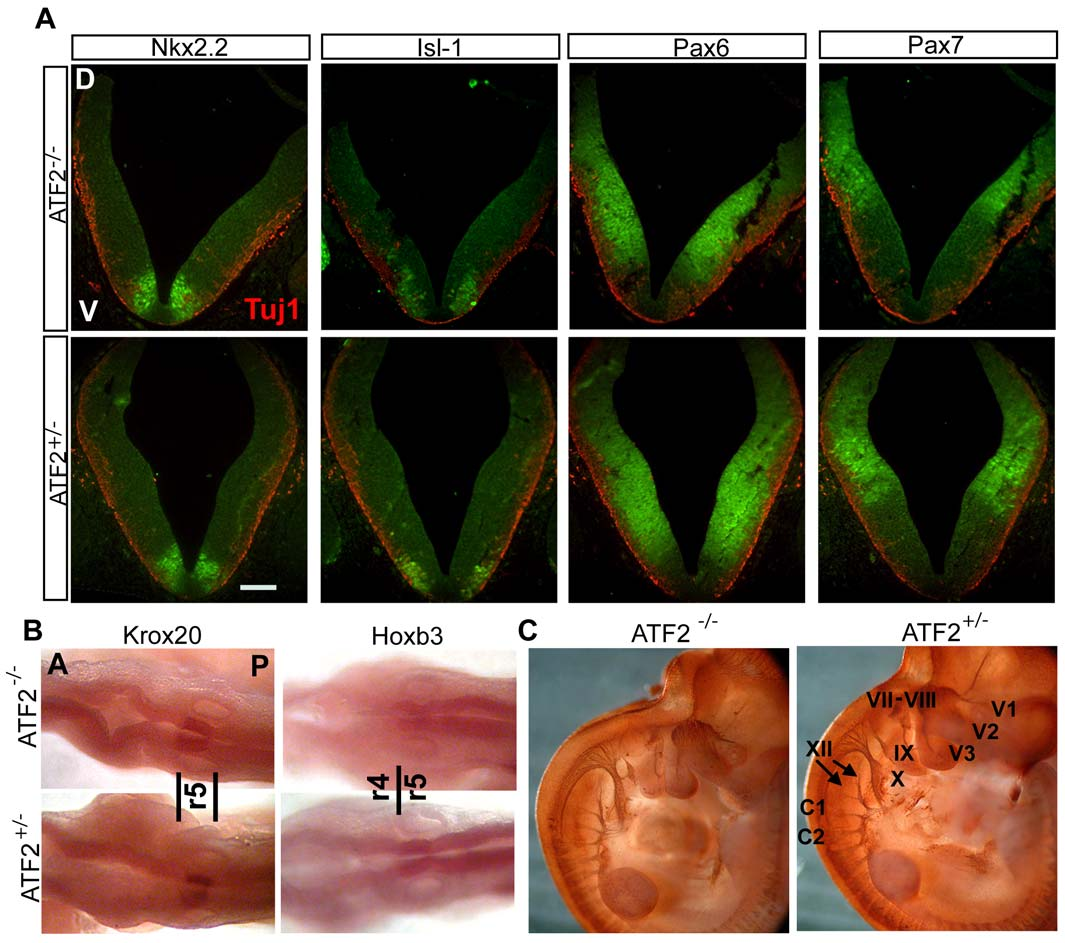
Dorso-ventral (D–V) and antero-posterior (A–P) patterning is normal in *Atf2* *^−^* ^*/−*^ brainstem at E10.5. (A) Double immunofluorescence staining for class III β-tubulin (Tuj1, red) and for neuronal class progenitor cells markers Nkx2.2, Pax6, Pax7 (all green) and postmitotic marker Isl-1 (green) in *Atf2^−/−^* and *Atf2^+/−^* brainstem. No significant difference was observed in the expression domains of the transcription factors between the two genotypes. Bar: 100 µm. (B) Whole-mount *in situ* hybridization for Krox20 and Hoxb3. No differences were observed in the expression domain between the two genotypes. r4, r5, rhombomere 4 and 5. Note that, at this stage, Krox20 expression is seen in rhombomere 5 (r5) only. (C) *Atf2^−/−^* and *Atf2^+/−^* whole-mount immunostaining with NF-M antibody at E12.5. Fiber nerves extend normally from *Atf2^−/−^* hypoglossal (XII) and vagal (X) nuclei. C1, C2, first and second cervical nerve, XII, hypoglossal nerve, X, IX, vagal and glossopharyngeal nerves, VII, VIII, acousticofacial nerve, V3, mandibular nerve, V2, maxillary nerve, V1, ophthalmic nerve.

To identify the causes leading to the cytological and morphological defects of the *Atf2^−/−^* brainstem at E18.5, we analysed the brainstem at earlier developmental stages. For this, we initially focused on the generation of progenitor cells along the dorsoventral axis at E10.5. The generation of these progenitor cells has been thoroughly studied *in vivo* and involves a number of transcription factors, including Pax6, Pax7, and Nkx2.2, which specify the identity of several classes of neurons [24]. When we compared ATF2 knockout and heterozygous embryos for the generation of progenitor cells at E10.5, we did not observe any significant differences in the expression patterns of these transcription factors (Figure 3A). We then analysed the antero-posterior expression pattern of two genes, *Krox20* and *Hoxb3*, known to play an important role in caudal hindbrain segmentation [25], [26]. Whole-mount *in situ* hybridization did not reveal any differences in the expression pattern of Krox20 and Hoxb3 between *Atf2^−/−^* and *Atf2^+/−^* embryos (Figure 3B). Motoneurons also extended their axons normally, as shown by embryo whole-mount immunostaining of neurofilament M (NF-M) at E12.5 (Figure 3C). These results suggest that ATF2 is not essential for motoneuron generation but is likely to play a role later in development.

### ATF2 is expressed in newly-born somatic and visceral motoneurons of the brainstem

**Figure 4:**
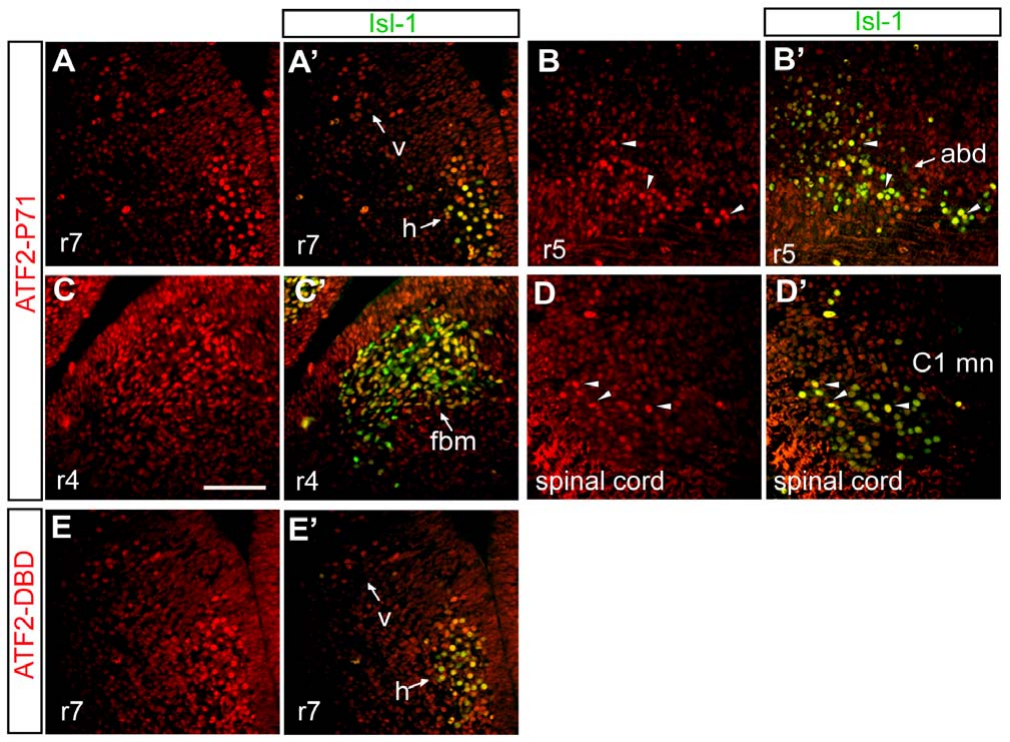
ATF2 expression in somatic and visceral motoneurons at E11.5 and E12.5. (A–D) Fluorescence immunostaining for phosphorylated ATF2 (ATF2-PT71, red). (A'–D') Double immunofluorescence staining for phosphorylated ATF2 (red) and postmitotic marker Isl-1 (green). Phosphorylated ATF2 is robustly detected in E12.5 hypoglossal (h) and dorsal vagal (v) motoneurons (A, A'), in E12.5 abducens (abd) motoneurons (B, B', arrowheads) and in facial branchiomotor neurons (C, C') at E11.5. In addition phospho-ATF2 is also detected in C1 motoneurons of the spinal cord at E13.5 (D, D', arrowheads). Little or no expression of phosphorylated ATF2 is found in the surrounding cells or on the dorsal side of the brainstem at this stage. Bar: 100 µm. (E, E') E12.5 hypoglossal (h) and dorsal vagal (v) motoneurons express ATF2 as shown by immunostaining with ATF2-DBD antibody (red) and Isl-1 (green). r4, r5, r7, rhombomere, 4, 5, and 7.

To understand ATF2 expression patterns in developing motoneurons, we stained brainstem sections for ATF2 at E11.5–E12.5. For this we used antibodies directed against its DNA-binding domain (ATF2-DBD) as well as antibodies that recognize phosphorylated ATF2 at Thr71 (ATF2-PT71). Both of these antibodies were highly specific for their respective epitopes as shown in Figure S2. This analysis revealed that ATF2 is robustly expressed in the majority of motoneurons that were identified by co-staining with the motoneuron-specific marker Isl-1. Specifically, ATF2 as well as phosphorylated ATF2 were detected in hypoglossal, dorsal vagal and abducens motoneurons at E12.5 (Figure 4A and A', 4B and B', 4E and E') and in facial branchiomotor neurons at E11.5 (Figure 4C and C'). We did not find significant expression of ATF2 or ATF2-PT71 in other neurons of the brainstem at these developmental stages (data not shown). Interestingly, ATF2-PT71 is also observed in motoneurons of the spinal cord at the C1 level (Figure 4D and D'), suggesting that ATF2 may play a role in the spinal cord, in addition to the brainstem. These results show that ATF2 is specifically expressed, between E11.5 and E12.5, in all visceral and somatic motoneurons we examined and may thus play a role in the further differentiation of these neurons.

### Survival of hypoglossal and abducens motoneurons is dependent on ATF2 expression

**Figure 5:**
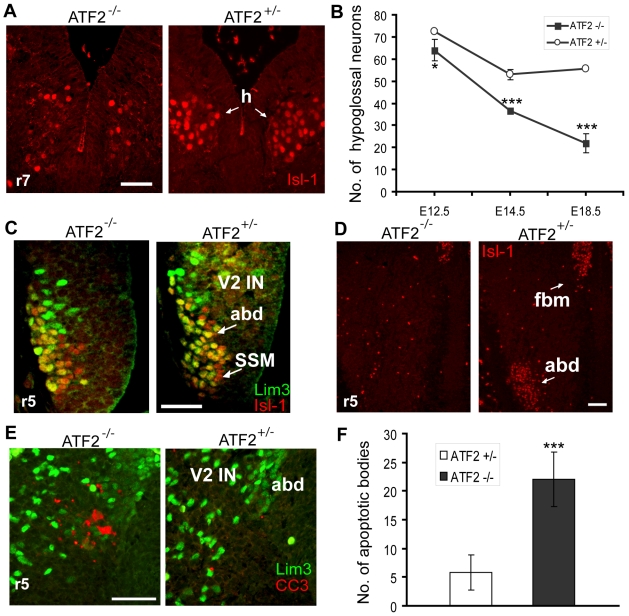
Somatic motoneuron defects in *Atf2* *^−^* ^*/−*^ embryos. (A) Isl-1 immunostaining (red) of *Atf2^−/−^* and *Atf2^+/−^* hypoglossal motoneurons at E14.5 reveal the reduction of Isl-1 positive hypoglossal motoneurons in *Atf2^−/−^* embryos. (B) Quantitative analysis of hypoglossal motoneurons (mean number per section ± SD) in serial transversal sections of rhombomere 7 (r7) by Isl-1 immunostaining for E12.5 and E14.5 embryos or by 5-HT immunostaining and hematoxylin counterstaining for E18.5 embryos. Student's two-tailed t-Test, *, p = 0.04; ***, p<0.01; ***; p<0.001. (C) Coimmunostaining of abducens motoneurons with Isl-1 (red) and Lim3 (green) antibodies reveals normal production of these motoneurons in *Atf2^−/−^* mice at E10.5 in r5. V2 IN, V2 interneurons, abd, abducens motoneurons, SSM, superior salivatory motoneurons. (D) Complete loss of abducens motoneurons (abd) in *Atf2^−/−^* E12.5 embryos as revealed by Isl-1 (red) immunostaining. fbm, facial branchiomotor neurons. (E) Coimmunostaining of abducens motoneurons with Cleaved Caspase 3 (CC3, red) and Lim3 (green) antibodies reveals increased apoptosis in *Atf2^−/−^* motoneurons at E11.5. Lim3 strongly labels V2 interneurons and more weakly abducens motoneurons. Bar: 100 µm. (F) Quantitative analysis of apoptotic bodies (mean number per section ± SD) in serial transversal sections of rhombomere 5 at E11.5 reveals increased apoptosis of abducens neurons in *Atf2^−/−^* embryos. ***, p<0.01.

We next investigated the importance of ATF2 in later motoneuron development. Since we have previously found a reduction in hypoglossal motoneurons at E18.5, we examined the specification and further development of these motoneurons in *Atf2^+/−^* and *Atf2^−/−^* embryos. For this we stained serial transversal sections of rhombomere 7 with the postmitotic marker Isl-1 and counted the number of Isl-1^+^ hypoglossal neurons present at E12.5 and E14.5. This staining and, in addition, staining of motoneurons with 5-HT (see Materials and Methods for quantification) at E18.5 revealed a significant and progressive loss of hypoglossal motoneurons in ATF2 mutant embryos (Figure 5A and B). At E12.5, motoneurons of the abducens nucleus appeared to be entirely absent in *Atf2^−/−^* embryos (Figure 5D), although they were normally produced in rhombomere 5 at E10.5 (Figure 5C). One possibility is that the loss of these neurons was caused by increased apoptosis. Immunostaining with the apoptotic marker Cleaved Caspase 3 revealed a significant increase in apoptotic bodies in *Atf2^−/−^* compared to *Atf2^+/+^* abducens motoneurons (Figure 5E and F). However, in contrast, no significant increase in Caspase dependent, apoptotic cell death was detected in hypoglossal motoneurons (data not shown). These results suggest that ATF2 has a strong pro-survival role, and its absence leads to Caspase 3 dependent cell death at least in early-born abducens neurons. However, the gradual disappearance of *Atf2^−/−^* hypoglossal neurons appears to be independent of Caspase 3 activity.

### *Atf2^−/−^* somatic and visceral motoneurons express hyperphosphorylated JNK and c-Jun

**Figure 6:**
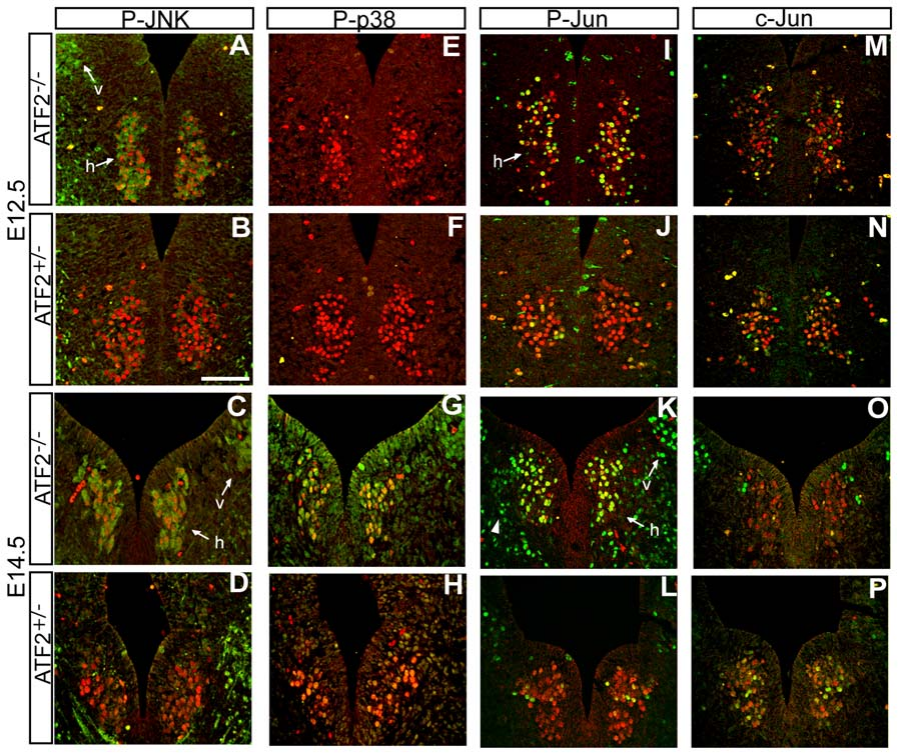
Enhanced phosphorylation of JNK, p38, and c-Jun in *Atf2* *^−^* ^*/−*^ motoneurons. Double immunofluorescence staining of Isl-1 (A–P, red) and phospho-JNK (Thr183/Tyr185, green, A–D), phospho-p38 (Thr180/Tyr182, green, E-H), phospho-c-Jun (Ser73, green, I-L) and pan-c-Jun (green, M–P) in hypoglossal (h) and vagal (v) motoneurons at E12.5 and E14.5. (A–D) JNK is hyperphosphorylated in both motoneuron types in *Atf2^−/−^* (A and C) compared to *Atf2^+/−^* (B and D) embryos at E12.5 and E14.5. (E–H) Phosphorylation of p38 is hardly detectable in *Atf2^+/−^* and *Atf2^−/−^* at E12.5 (E and F) but significantly increases at E14.5 in motoneurons of *Atf2^−/−^* (G) compared to *Atf2^+/−^* embryos (H). (I-L) c-Jun is hyperphosphorylated in motoneurons of *Atf2^−/−^* (I and K) compared to *Atf2^+/−^* (J and L) embryos at E12.5 and E14.5. Strong phosphorylation of c-Jun is notable in hypoglossal and vagal motoneurons at E14.5 and there is a weaker but detectable phosphorylation signal in surrounding cells in *Atf2^−/−^* embryos (K, arrowheads). (M, N) High and moderate levels of c-Jun were observed in hypoglossal motoneurons but were indistinguishable between *Atf2^−/−^* and *Atf2^+/−^* embryos at E12.5. (O, P) At E14.5, *Atf2^−/−^* hypoglossal neurons tend to express c-Jun at lower levels than in *Atf2^+/−^* neurons; notably some strong c-Jun labeling was detected in some Isl-1 negative cells in *Atf2^−/−^*. Bar: 100 µm.

To understand the mechanism by which cell death is induced in *Atf2^−/−^* hypoglossal motoneurons, we analysed signaling pathways that could be involved. Activation of the stress activated MAP kinase JNK and of its target substrate c-Jun are important mediators of neuronal stress response after cerebral ischemia and central nerve fiber tract transection [27]. Enhanced c-Jun expression also occurs in neurodegeneration disorders [28]. We therefore investigated whether ATF2 could regulate the activation of stress activated kinases in motoneurons. Staining with antibodies against phosphorylated (Thr183/Tyr185) JNK revealed that P-JNK was significantly increased in hypoglossal and dorsal vagal *Atf2^−/−^* motoneurons at E12.5 compared to *Atf2^+/−^* motoneurons (Figure 6A and B). Enhanced phosphorylation of JNK was also detected in *Atf2^−/−^* facial branchiomotor neurons (Figure S3E and F) and in *Atf2^−/−^* motoneurons of the spinal cord at the C1 level (Figure S3I-L). JNK hyperphosphorylation in *Atf2^−/−^* was also maintained at later stages in hypoglossal and vagal motoneurons at E14.5 (Figure 6C and D). Importantly, JNK phosphorylation was also increased in hypoglossal and vagal motoneurons of *Atf2^AA^* embryos, similar to the results observed in ATF2 knockout brainstems (Figure S3A–D). Interestingly, while levels of P-p38 (Thr180/Tyr182) were very low in both knockouts and heterozygotes at E12.5 (Figure 6E and F), at E14.5 a significant increase in the phosphorylation of p38 was detected in *Atf2^−/−^* motoneurons but not in controls (Figure 6G and H). No significant differences were found in the phosphorylation status of ERK1/2 at E12.5 or E14.5 (Figure S3M-P).

**Figure 7:**
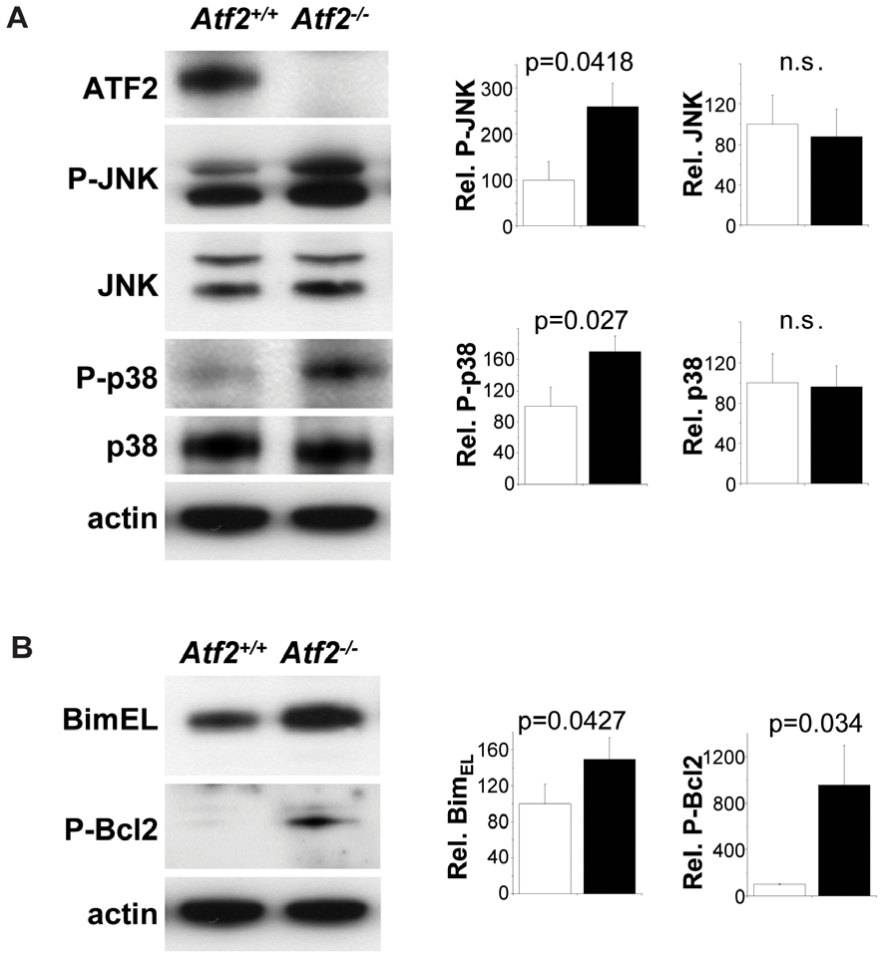
Up-regulation of P-JNK and P-p38 in *Atf2* *^−^* ^*/−*^ hindbrains. (A) Western blot analysis shows increased P-JNK (Thr183/Tyr185) and P-p38 (Thr180/Tyr182) in *Atf2^−/−^* compared to *Atf2^+/+^* E14.5 hindbrains extracts. (B) Expression levels of Bim_EL_ and P-Bcl2 (Ser70) are shown. Bar graphs show relative signal intensity between *Atf2^+/+^* (white bars) compared to *Atf2^−/−^* (black bars). Significance values (p) were determined from 3 independent samples using Student's t-test.

Subsequently we performed Western blot analysis of extracts derived from hindbrain regions of E14.5 embryos. These confirmed that both P-JNK and P-p38 were up-regulated in *Atf2^−/−^* samples compared to controls (Figure 7A). In addition we found significantly increased levels of Bim_EL_ and P-Bcl2 (Ser70) (Figure 7B). The phosphorylation of Ser70 leads to inactivation of Bcl2 and promotes apoptosis. This result is therefore consistent with the observed increased apoptosis in mutant neurons.

### ATF2 regulates the expression of c-Jun and dual specificity phosphatase genes

Since c-Jun is a substrate of JNK activity it is conceivable that high levels of P-JNK would also lead to increased levels of phosphorylated c-Jun. Indeed, phosphorylation of c-Jun at Ser73 was found significantly increased in *Atf2^−/−^* hypoglossal neurons at E12.5 and E14.5 (Figure 6I-L) as well as in facial branchiomotor neurons (Figure S3G and H). While phosphorylation of c-Jun was strongest in cranial nerve nuclei, enhanced phosphorylation was also observed in many cells in the mantle zone at E14.5 (Figure 6K, arrowheads, and data not shown). Therefore ATF2 is essential to regulate c-Jun activity in developing motoneurons possibly through restricting the activities of JNK. ATF2 can also regulate the expression of c-Jun. To test whether this is the case in neurons of the brainstem we immuno-stained sections for pan-c-Jun. At E12.5, comparable expression patterns of c-Jun were observed between *Atf2^−/−^* and *Atf2^+/−^* hypoglossal neurons (Figure 6M and N). However, at E14.5, c-Jun expression was significantly decreased in *Atf2^−/−^* hypoglossal neurons compared to heterozygous control embryos, while retaining strong expression in some neurons of the vagal nuclei which were otherwise negative for Isl-1 (Figure 6O and P). From these results we concluded that c-Jun has a complex expression pattern in developing motoneurons, and is, at least, partly under ATF2 transcriptional regulation.

**Figure 8:**
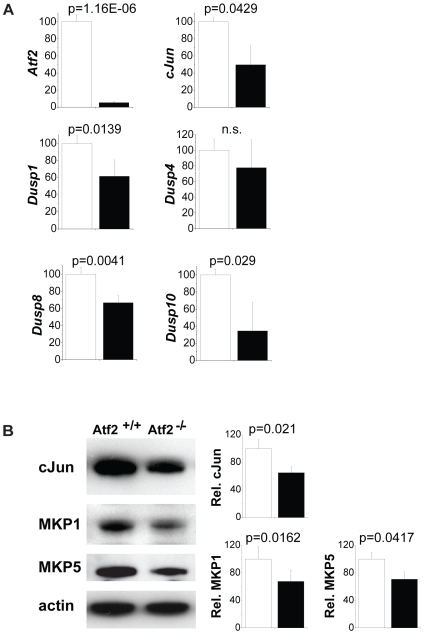
Expression of ATF2 target genes in hindbrain motoneurons. Real-time quantitative PCR assays of laser micro-dissected tissue of E14.5 hindbrains. Bar graphs show relative expression values between *Atf2^+/+^* (white bars) and *Atf2^−/−^* (black bars) for Atf2, c-Jun, Dusp1, Dusp4, Dusp8, and Dusp10 mRNAs. Significance values (p) were determined from 3 independent samples using Student's t-test.

To confirm that c-Jun dependence on ATF2 is at the level of mRNA expression we isolated RNA, using laser capture micro-dissection, from regions of the hindbrain of E14.5 embryos that correlated with high Isl-1 expression. Quantitative mRNA analysis revealed significant loss of c-Jun mRNA in *Atf2^−/−^* neurons (Figure 8A). This observation was also confirmed by RNA *in situ* hybridization using a c-Jun antisense mRNA probe (Figure S4) and by Western blot which shows a decrease in c-Jun present in mutant neurons (Figure 8B).

In addition to c-Jun we also analysed the expression of genes encoding dual specificity phosphatases, which have been shown previously to be targets of ATF2 in the embryonic liver. We found reduced expression of Dusp1, Dusp8, and Dusp10 in *Atf2^−/−^* neurons whereas other Dusp genes, e.g. Dusp4 and Dusp6 (data not shown) showed similar expression levels between wild-types and knockouts (Figure 6A). Reduced expression of MKP1 (the gene product of *Dusp1*) and MKP5 (the gene product of *Dusp10*) was also confirmed by Western blot (Figure 8B). These data therefore suggest that, in developing neurons, ATF2 is required for the expression of specific phosphatases that can negatively regulate the activities of JNK and p38.

### Newly-born *Atf2^−/−^* motoneurons accumulate neurofilament aggregates

**Figure 9:**
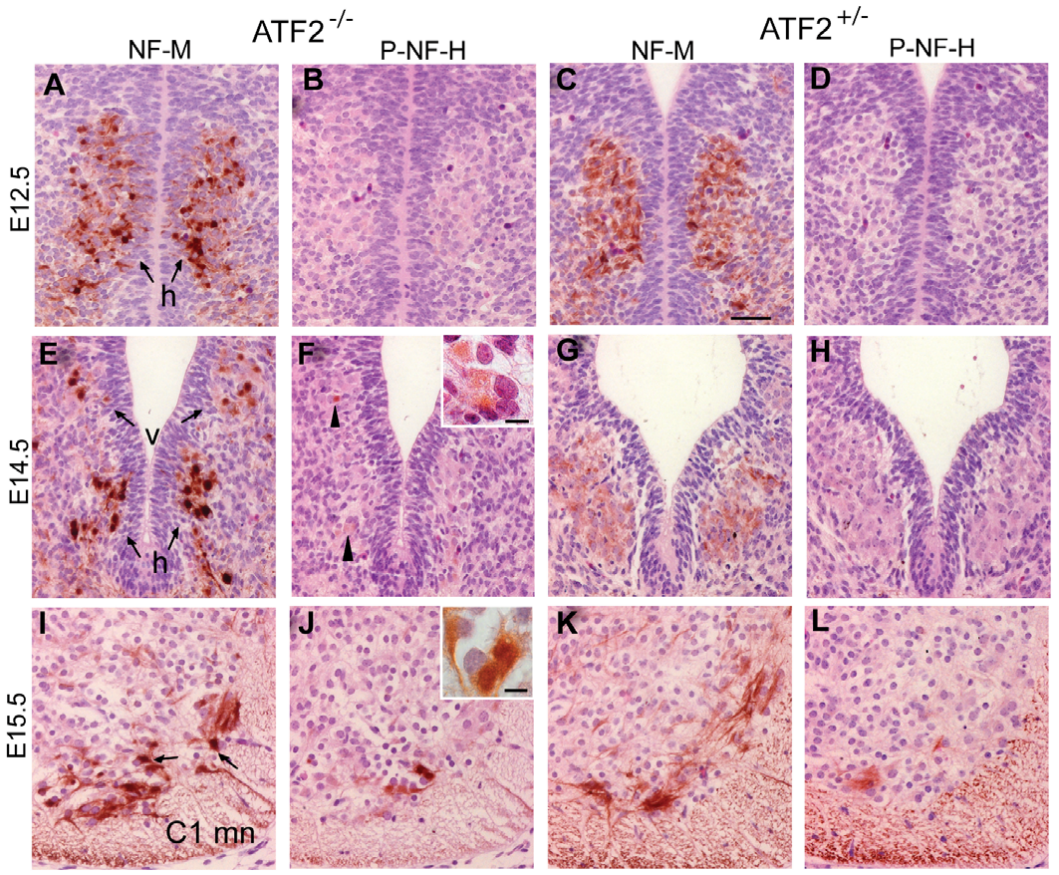
Neurofilament M (NF-M) accumulation in *Atf2* *^−^* ^*/−*^ motoneurons. (A–D) Immunostaining against NF-M (A and C) and phosphorylated NF-H (B and D) at E12.5 reveals strong accumulation of NF-M in the soma of *Atf2^−/−^* hypoglossal motoneurons (h) but not in *Atf2^+/−^* motoneurons (compare A and C). Phosphorylation of NF-H as detected with the antibody RMO24.9 was not yet observed at this age. (E–H) Immunostaining against NF-M (E and G) and phosphorylated NF-H (F and H) at E14.5 reveals strong accumulation of NF-M in hypoglossal (h) and vagal (v) motoneurons in *Atf2^−/−^* (E) compared to *Atf2^+/−^* embryos (G). Phosphorylation of NF-H can be detected in *Atf2^−/−^* motoneurons (F, arrowheads) but not in *Atf2^+/−^* neurons (H). Inset: magnification of P-NF-H stained hypoglossal neurons. (I-L) Immunostaining against NF-M (I and K) and phosphorylated NF-H (J and L) at E15.5 reveals aberrant accumulation of NF-M and hyperphosphorylation of NF-H in the soma of *Atf2^−/−^* spinal cord motoneurons at the C1 level (C1 mn) but not in *Atf2^+/−^* neurons. Bar: 100 µm. Inset: magnification of P-NF-H stained C1 motoneurons. Bar: 25 µm.

JNK activity has been associated with cytoskeletal integrity [29]. Since significantly higher levels of P-JNK were detected in the cytoplasm of ATF2 mutant motoneurons, we analysed the neurofilament cytoskeleton in newly-born motoneurons. Immunostaining with antibodies against NF-M and phospho-NF-M revealed that the cell bodies of E12.5 *Atf2^−/−^* hypoglossal neurons aberrantly accumulate NF-M compared to the more diffuse pattern seen in *Atf2^+/−^* (Figure 9A and C). At E12.5, phosphorylated NF-M was not yet detected. We further show that NF-M aggregates are also present in E14.5 *Atf2^−/−^* hypoglossal and vagal motoneurons somas (Figure 9E and G). At this age, we also detected phosphorylated NF-H strongly in *Atf2^−/−^* but only weakly in *Atf2^+/−^* motoneurons (Figure 9F and H). Accumulation of NF-M as well as P-NF-H was also observed in the anterior horn of *Atf2^−/−^* spinal cords at E15.5 (Figure 9I-L). These results indicate that neurofilament cytoskeleton integrity is abnormal in post-mitotic *Atf2^−/−^* motoneurons. This phenotype coincides with the appearance of hyperphosphorylated JNK and p38. Hyper-activation of the stress activated protein kinase pathways may therefore be critical for motoneuron degeneration and other pathological lesions observed at E18.5.

## Discussion

### Neuronal-specific ATF2 expression is required for embryonic survival

The study presented here reveals novel, essential functions for ATF2 in motoneuron integrity during mammalian development and provides a mechanistic insight into the regulation of stress kinase signaling in the central nervous system. Previous phenotypic studies on ATF2 hypomorphic alleles indicated a range of neurological abnormalities in the cerebellum as well as vestibular sense organs [11] of adult mice. More recent analyses have also uncovered defects in catecholaminergic neurons of the pons and the medulla [20]. Together these findings suggest a fundamental role for ATF2 in neurological functions in adult mice. In the present work, we demonstrate that ATF2 has much earlier roles in CNS development and which become critical for survival at the end of foetal development. To show this we developed neuronal specific ATF2 knockouts by crossing *nestin-Cre* transgenic with ATF2 floxed animals. Surprisingly, the neural specific deletion of ATF2 resulted in complete perinatal lethality due to respiratory defects, comparable to the phenotype of the *Atf2^−/−^* or *Atf2^AA^* mutants. Therefore, an early requirement of ATF2 in the central nervous system is critical for postnatal viability. Previously, it has been suggested that the lethality observed in *Atf2^0/0^* mice was a result of defective placenta development [12]. However, the ATF2 mutants produced in our lab do not support this notion. Firstly, analysis of *Atf2^−/−^* embryos and newborns did not reveal any observable morphological defects in the placenta [13]. Secondly, the *meox2-Cre;Atf2^f/f^* conditional allele of ATF2 which targets the epiblast, but not the placenta, also leads to perinatal lethality therefore excluding placental defects as an underlying cause. Thirdly, the *nestin-Cre;Atf2^f/f^* derived conditional allele of ATF2 phenocopies most if not all of the neuronal defects that were uncovered in the *Atf2^−/−^* mutants.

Analysis of ATF2 mutant brains has revealed a number of anatomical defects in the CNS. At E18.5, brainstem and the cerebellum were significantly reduced in size. In addition, defects in the laminar patterning of the cerebellum, and enlargement of the central canal were apparent. In further detailed analyses we found that several cranial motor nuclei were strongly affected in mutant brainstems. In particular, cranial motoneurons of the facial, hypoglossal, abducens and dorsal vagal nuclei were significantly reduced in numbers or severely degenerated. Can individual or combinations of these defects explain the early postnatal respiratory disorder and death of ATF2 mutant mice? Mouse knockouts of several transcription factors such as MafB, Phox2a, Phox2b, Rnx, and Mash-1, have been shown to affect different groups of respiratory neurons in the brainstem resulting in respiratory phenotypes and death at or shortly after birth [30]. These phenotypes included loss of neurons in the (nor)adrenergic and serotonergic centers [31], in the nucleus tract solitarius [32] and in the prebötzinger complex [33]. However, none of these appear to affect cranial motoneurons. Cranial motoneurons innervate muscles controlling airway dimensions and exhibit an important rhythmic respiratory activity that is synchronous with the activity of respiratory neurons of the medulla during expiration and inspiration [34]. Importantly, in humans, hypoplasia of several brainstem nuclei affected in our ATF2 mutant mice, including the inferior olivary nuclei, the facial, the dorsal vagal and the hypoglossal nuclei have been reported in cases of intrauterine or neonatal sudden death as well as in sudden infant death syndrome [35]. Interestingly, cranial motoneuron nuclei hypo-development is frequently associated with pulmonary hypoplasia and aspiration pneumonia [35], [36]. It is therefore plausible that the brainstem malformation caused by ATF2 mutation leads to similar respiratory defects to those seen in some forms of human sudden death syndromes.

### ATF2 has a strong pro-survival role in somatic motoneurons of the brainstem

In order to explain the motoneuron phenotype observed at E18.5, we sought to determine the earliest developmental events for which neuronal ATF2 functions may be critical. At around E10.5, neuronal progenitors receive positional information along the anteroposterior and dorsoventral axes, and a number of homeodomain proteins are key molecules for this specification [37]. Notably, vagal motoneurons differentiate ventrally within a domain in which progenitor cells express Nkx2.2, whereas hypoglossal motoneurons differentiate dorsally within a domain in which progenitors express Pax6 [38]. Several mouse knockouts for these homeobox genes show changes in the fate of motoneurons, alterations in the migratory behavior of motoneurons, and defects in motor axon extension [24], [39], [40], [41]. In addition, gene inactivation of *Krox20* and *Hoxb3*, which control the assignment of specific rhombomere identities, both lead to the complete elimination of abducens motoneurons as well as defects in other motoneuron populations [42], [43]. While germ line deletion of *Hoxb3* results in viable animals, *Krox20* mutant offspring display significant respiratory defects and a large proportion die within hours after birth [44], [45]. At E10.5, dorsoventral patterning proceeded normally in ATF2 mutants as judged by the expression pattern of Nkx2.2, Isl-1, Pax6 and Pax7 which was similar to that in heterozygotes. Krox20 and Hoxb3 were also both normally expressed in the knockouts, suggesting that rhombomere 5 was intact. We also show normal extension of the hypoglossal, dorsal vagal and spinal cord motoneuron axons in ATF2 mutant embryos. In addition, Isl-1 staining revealed that all major somatic and viscerobranchiomotor neurons, including abducens, hypoglossal, trigeminal (data not shown), facial and dorsal vagal motoneurons were produced. Therefore, ATF2 inactivation does not appear to affect early anteroposterior and dorsoventral cell specification.

ATF2 and phosphorylated ATF2 proteins are specifically and robustly expressed in newly-born hypoglossal, dorsal vagal, facial and abducens motoneurons between E11.5 and E12.5 as well as in the spinal cord in C1 motoneurons at E13.5. This expression pattern suggests a functional involvement of ATF2 in motoneuron development. Indeed, inactive ATF2 leads to the complete loss of abducens motoneurons at E11.5, as well as the gradual loss of hypoglossal motoneurons between E12.5 and E18.5. At least in the case of abducens motoneurons we could demonstrate that cell loss is due to aberrant caspase- dependent apoptosis. In contrast, loss of hypoglossal neurons appears to be Caspase 3-independent and it remains to be seen which cell death mechanism is responsible. These results however suggest that ATF2 is an important suppressor of developmental cell death and its loss leads to degeneration of somatic motoneurons.

### Loss of functional ATF2 leads to hyperphosphorylated JNK and p38, and results in somatic and visceral motoneuron degeneration

In exploring a molecular mechanism underlying the observed neurodegeneration we found that JNK and c-Jun were hyperphosphorylated, and therefore, hyperactive in cranial motoneurons from as early as E12.5, and p38 from E14.5. Lasting N-terminal phosphorylation of c-Jun and JNK has been associated with cerebral ischemia-reperfusion and nerve fiber transection [27]. In addition, phosphorylation of c-Jun was shown to be required for embryonic programmed cell death in motoneurons in mouse and chick [46]. Furthermore, JNK dependent phosphorylation of c-Jun is required for stress induced apoptosis in hippocampal and cortical neurons [47]. It could therefore be possible that in ATF2 mutant embryos JNK-activated c-Jun leads to cell loss of motoneurons by apoptosis. However, not all the neuronal cell loss we observe is via caspase dependent apoptosis which c-Jun may be capable of inducing. Furthermore, after crossing ATF2 mutant mice with c-Jun mutant mice which are deficient in phosphorylaton by JNK [47] the early postnatal lethal phenotype of the ATF2 single mutant prevailed (unpublished results), suggesting that the observed neuronal cell death is probably independent of JNK dependent c-Jun activation. Indeed, JNK has been shown to phosphorylate a number of apoptotic regulators of the Bcl2 family, including Bcl2 [48], [49], Bclx(L) [50], and Bim [51].

The observed high levels of active JNK, as well as p38, may have roles in inducing neuronal cell death independent of c-Jun. This possibility is emphasized by the higher cytoplasmic levels of phospho-JNK observed in mutant motoneurons (see Figure 6). The hyperphosphorylation of JNK, and p38, coincided with the accumulation, and phosphorylation, of neurofilament proteins specifically in motoneurons but not in other subpopulations of neurons (e.g. interneurons, or sensory neurons). Studies in neuroblastoma cells have shown that MAP kinase ERK is involved in normal neurofilament NF-H phosphorylation whereas neurotoxin induced JNK activation leads to aberrant NF-H phosphorylation and accumulation [52], [53]. In addition, Ackerley et al. have shown that p38α phosphorylates NF-M and NF-H on their side-arm domains in cortical neurons, thereby modulating the flexibility and stability of the cytoskeleton [29], [54]. Abnormal accumulations of neurofilaments with phosphorylated NF-H side arms are pathological features of motoneuron diseases such as amyotrophic lateral sclerosis (ALS). Particularly, enhanced p38 activation has been reported in motoneurons from transgenic mice expressing ALS-linked SOD1 mutants [55], [56], [57] as well as in human ALS patients [54].

In addition to abnormal accumulation and phosphorylation of neurofilaments in cell bodies and proximal axons we observed other histopathological features of motoneuron degeneration, including ballooned perikarya and vacuolization. Vacuolization is a further prominent feature of the ALS-model SOD1 mutant mice [58]. Results in our mutants show that these vacuoles may primarily contain lipids as revealed by Sudan black B staining. This coincides with increasing evidence of abnormalities in lipid metabolism in ALS patients and lipids observed in SOD1 mutant neurons [23], [59]. In other studies of ALS linked SOD1 mutant mice activated JNK has also been observed [60]. In addition, pathological roles for JNK activation have been well documented in other neurodegenerative disorders such as Parkinson's disease and Alzheimer's disease [61], [62].

From our study, it is not yet clear how ATF2 deficiency leads to dysregulated JNK and p38 activities. One possibility is that ATF2 mutant neurons display an aberrant response to stress conditions such as oxidative stress, or inflammatory stress, for which ATF2 may have important roles [63]. Stressed neurons may lead to high levels of active stress kinases which may be dangerous to cell survival. Alternatively, a more direct role for ATF2 in stress kinase regulation was uncovered in our analysis of ATF2 functions for the survival of liver cell precursors [13]. Here, loss of functional ATF2, and its closest homologue ATF7, resulted in hyperactive p38 kinase and p38-induced apoptosis in the embryonic liver cells concomitant with reduced levels of members of the DUSP family of MAP kinase phosphatases. A similar defect in a negative feedback regulation of stress kinases may operate in ATF2 single mutant mice in specific regions of the developing brain. In conclusion, our observations demonstrate that MAP kinase activities require tight control during neuronal development and that ATF2 is both a target and a regulator of these activities.

## Materials and Methods

### Generation of ATF2 mutant transgenic mice

*Atf2^−/−^*, *Atf2^AA^*, and *Atf2^f/f^* mice were reported previously [13]. *Atf2^Δneuron^* mice were generated by crossing *Atf2^f/+^ nestin-Cre* mice with *Atf2^f/f^* mice. *Atf2^Δepiblast^* mice knockouts were produced by crossing *Atf2^f/+^ meox2-Cre* mice with *Atf2^f/f^* mice. The offspring genotypes were approximately of the expected frequency for all possible allele combinations. All animal work was approved by the Paterson Institute Ethical Review Committee and was performed within the limits of a license granted by the Home Office according to the Animals (Scientific Procedures) Act 1986.

### Immunohistochemistry

Brains were dissected out of embryos and were fixed overnight in 4% paraformaldehyde or frozen. Six-micron sections were produced from paraffin-embedded PFA-fixed samples or sucrose-embedded frozen samples. Immunohistochemistry was performed according to the antibody specification. For some antibodies, antigen retrieval was carried out in citrate buffer pH 6. For whole-mount immunostaining, E12.5 embryos were fixed in 100% methanol for two hours at 4°C and rehydrated in methanol/PBS/0.1% Tween-20 series. Embryos were washed in TBS pH 7.55/0.4% Triton X-100 and bleached by treatment with 2% H_2_O_2_ for 10 min. Embryos were then blocked in 5% non fat dry milk, incubated overnight with neurofilament M antibodies and revealed with Envision DAKO kit (Dako, K3955). Whole mount *in situ* hybridization was performed as previously described [64]. Krox20 and Hoxb3 RNA probes were kindly provided by Dr. F. Mechta-Grigoriou [65]. Sudan black B staining was performed according to histological techniques as described [66]. Transmission electron microscopy was performed according to standard procedures [67].

### Antibodies

Commercial primary antibodies used were anti-phospho-ATF2 (Thr71) (#9221, dilution 1∶100), anti-phospho-p44/p42MAPK (Thr202/Tyr204, #4376, 1∶100), anti-phospho-p38 MAPK (Thr180/Tyr182, #9211, 1∶100), anti-phospho-JNK (Thr183/Tyr185, #9251, 1∶100), anti-c-Jun (#9165, 1∶80) and anti-cleaved-caspase3 (Asp175, #9661, 1∶200), anti-Bim (C34C5, 1∶1000) all from Cell Signaling; anti-phospho-c-Jun (Ser73, #3502, Biovision, 1∶80); Nkx2.2, NF-M, Pax6, Pax7, Islet-1, Lim3 antibodies (concentrates, 1∶100) all from Developmental Studies Hybridoma Bank (Iowa, USA); anti-tyrosine hydroxylase (AB152, 1∶400), anti-choline acetyltransferase (AB144, 1∶100), anti-GFAP (AB5804, 1∶500), anti-calbindin D-28K (AB1778, 1∶500) all from Millipore; anti-5-HT (#11161, Progen, 1∶50); rabbit anti-Islet-1 (ab26122, Abcam, 1∶1000); anti-β-tubulin (Tuj1, MRB-435S, Covance); anti-MKP1 (sc-370), anti-MKP5 (sc-47663), anti-phospho-Bcl2 (Ser70, sc-47663) all from Santa Cruz Biotechnology. Anti-phospho-NF-M (clone RMO8, 1∶10) and anti-phospho-NF-H (clone RMO24.9, 1∶10) were a kind gift from Beat Riederer, (DBCM, University of Lausanne). ATF2-DBD antibodies were raised in rabbit against the peptide epitope RRAANEDPDEKRRK (BioGenes, Berlin) and affinity purified (Pharmacia).

### Cell counts

All counting and measurements were performed using the Neurolucida software. Isl-1^+^ hypoglossal cells were counted on transverse sections through the caudal medulla (r7–r8) of E12.5 and E14.5 heterozygous (n = 16 sections from two E12.5 embryos and n = 13 sections from two E14.5 embryos) and *Atf2^−/−^* mutants (n = 24 sections from three E12.5 embryos and n = 15 sections from two E14.5 embryos). E18.5 hypoglossal neurons were counted based on 5-HT immunostaining of 5-HT terminals in this nucleus as previously described [68] combined with hematoxylin staining of individual nuclei (n = 13 sections from two *Atf2^+/−^* embryos and n = −33 sections from four *Atf2^−/−^* embryos). Cleaved Caspase 3^+^ apoptotic cell bodies were counted on transverse sections of E11.5 *Atf2^+/−^* and *Atf2^−/−^* (n = 12 sections from three embryos for each genotype) and immunostained with Lim3 to identify abducens motoneurons.

### RNA and protein analysis

Protein was isolated from E14.5 hindbrains by extraction into Cytobuster reagent (Novagen). RNA was isolated from frozen E14.5 hindbrain sections using laser micro-dissection (LMD6000, Leica). Sections were immunostained with Isl-1 antibodies to identify motoneuron nuclei. Total RNA was isolated using RNAqueous-Micro Kit (Ambion). RNA quality was verified using RNA Nano Chips (Agilent) on Agilent 2100 Bioanalyser. RNA was amplified using Ovation Pico WTA System (Nugen). Real-time quantitative PCR assays were performed on ABI7900 (Applied Biosystems) using Jumpstart TaqReady Mix (Sigma) and gene specific primers and probes (Universal Probe Library, Roche).

## Supporting Information

Figure S1 **Hindbrain defects in E18.5 brain of ATF2 mutant embryos.** (A) Efficient deletion of *Atf2* floxed alleles by neuronal-specific Cre recombinase expression as revealed by HRP immunostaining using an antibody against the DNA-binding domain of ATF2. ATF2 is efficiently expressed in the hippocampus and the brainstem of *Atf2^f/+^;nestin-Cre* but is completely absent in *Atf2^f/f^;nestin-Cre*. (B) HRP immunostaining of calbindin in sagittal sections of cerebellum reveals lack of foliation and laminar distribution in *Atf2^AA^* and *Atf2^Δneuron^* mice compared to their control littermates. Bar: A, B, 250 µm.
(TIF)

Figure S2 **Epitope specificity of ATF2 antibodies.** (A-D') E11.5 transversal brainstem sections at the level of the facial branchiomotor neurons were fluorescently stained with antibodies against Isl-1 (green, A–D) and phospho-ATF2 at Thr71 (ATF2-PT71, red, A', B') or the DNA binding domain of ATF2 (ATF2-DBD, red, C', D'). ATF2-PT71 positive signals were found in *Atf2^+/AA^* (A') but not in *Atf2^AA^* neurons (B'). ATF2-DBD positive signals were found in *Atf2^+/+^* (C') but not in *Atf2^−/−^* neurons (D'). Bar: 100 µm.
(TIF)

Figure S3 **Hyperphosphorylation of JNK and c-Jun in ATF2 mutant motoneurons.** (A–D) Hyperphosphorylation of JNK (green; Isl-1, red) in hypoglossal (h) and vagal (v) motoneurons in *Atf2^AA^* embryos compared to control *Atf2^+/AA^* littermate at E12.5 (A and B) and E14.5 (C and D). (E–H) Hyperphosphorylation of JNK (E and F) and c-Jun (G and H) (green; Isl-1, red) in *Atf2^−/−^* facial branchiomotor neurons at E12.5. (I–L) Hyperphosphorylation of JNK (green, Isl-1 in red) in *Atf2^−/−^* C1 motoneurons of the spinal cord (arrowheads) at E13.5 (I and J) and E15.5 (K and L). (M–P) Fluorescence immunostaining of hypoglossal motoneurons against P-ERK1/2 (Thr202/Tyr204) (green) and Isl-1 (red). No differences were observed between *Atf2^−/−^* and *Atf2^+/−^* embryos at E12.5 (M and N) or E14.5 (O, P). Bar: 100 µm.
(TIF)

Figure S4 **Reduced expression of c-Jun in** ***Atf2*** *^−^* ^***/****−*^ **hindbrain neurons. **RNA *in situ* hybridization of c-Jun mRNA at E14.5. Overall signal intensity is stronger in *Atf2^+/+^* compared to *Atf2^−/−^*. Notably, staining of the hypoglossal nucleus (arrows) is markedly reduced in *Atf2^−/−^*. Bar: 100 µm.
(TIF)
