# Supplementary figures and images for: Loss of ATF2 Function Leads to Cranial Motoneuron Degeneration during Embryonic Mouse Development
Source: PLoS One. 2011 Apr 21;6(4):e19090. doi: 10.1371/journal.pone.0019090 (PMC3080913; doi:10.1371/journal.pone.0019090)

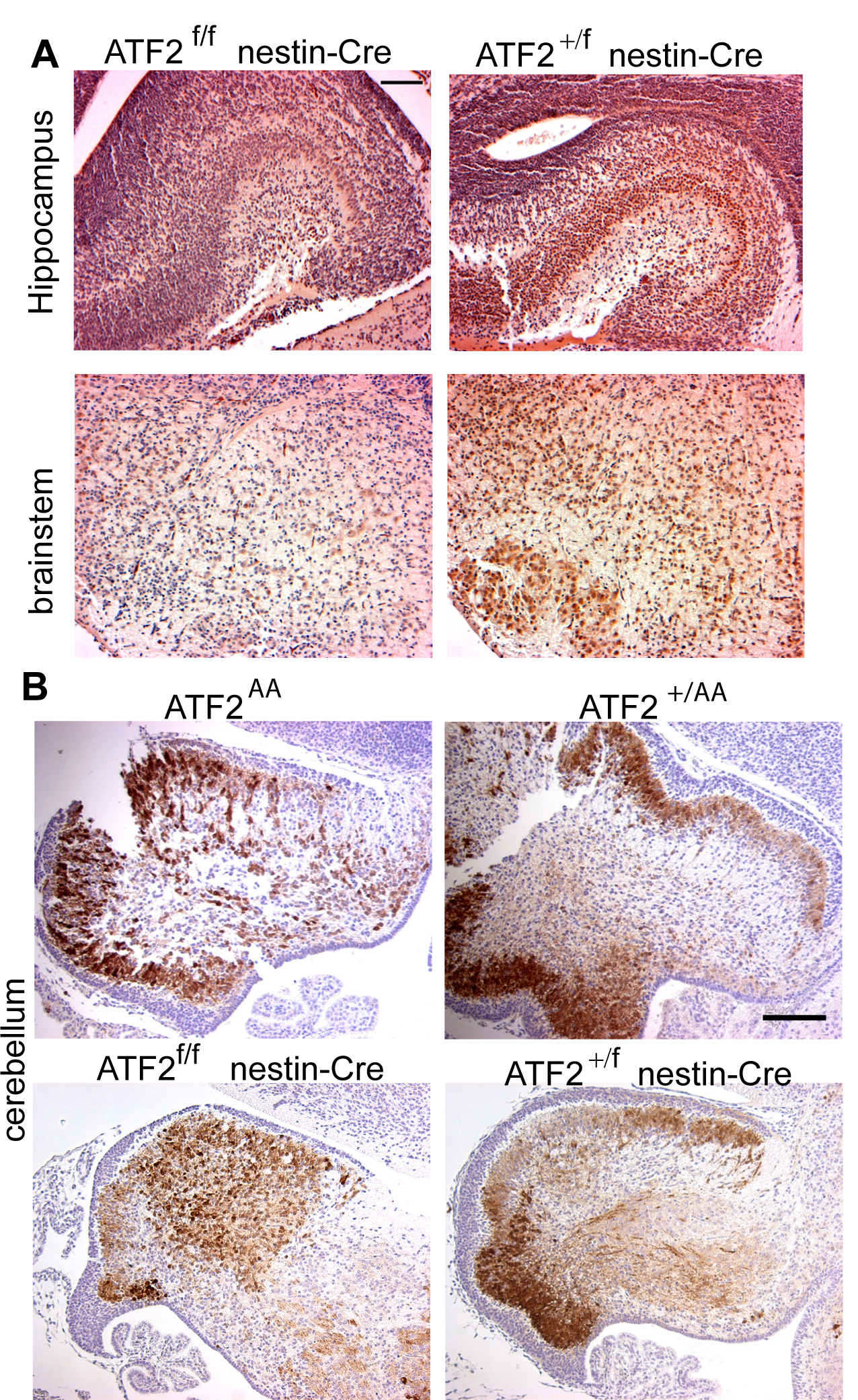

Supplement: Figure S1 — Hindbrain defects in E18.5 brain of ATF2 mutant embryos. (A) Efficient deletion of Atf2 floxed alleles by neuronal-specific Cre recombinase expression as revealed by HRP immunostaining using an antibody against the DNA-binding domain of ATF2. ATF2 is efficiently expressed in the hippocampus and the brainstem of Atf2f/+;nestin-Cre but is completely absent in Atf2f/f;nestin-Cre. (B) HRP immunostaining of calbindin in sagittal sections of cerebellum reveals lack of foliation and laminar distribution in Atf2AA and Atf2Δneuron mice compared to their control littermates. Bar: A, B, 250 µm. (TIF) [file pone.0019090.s001.tif]

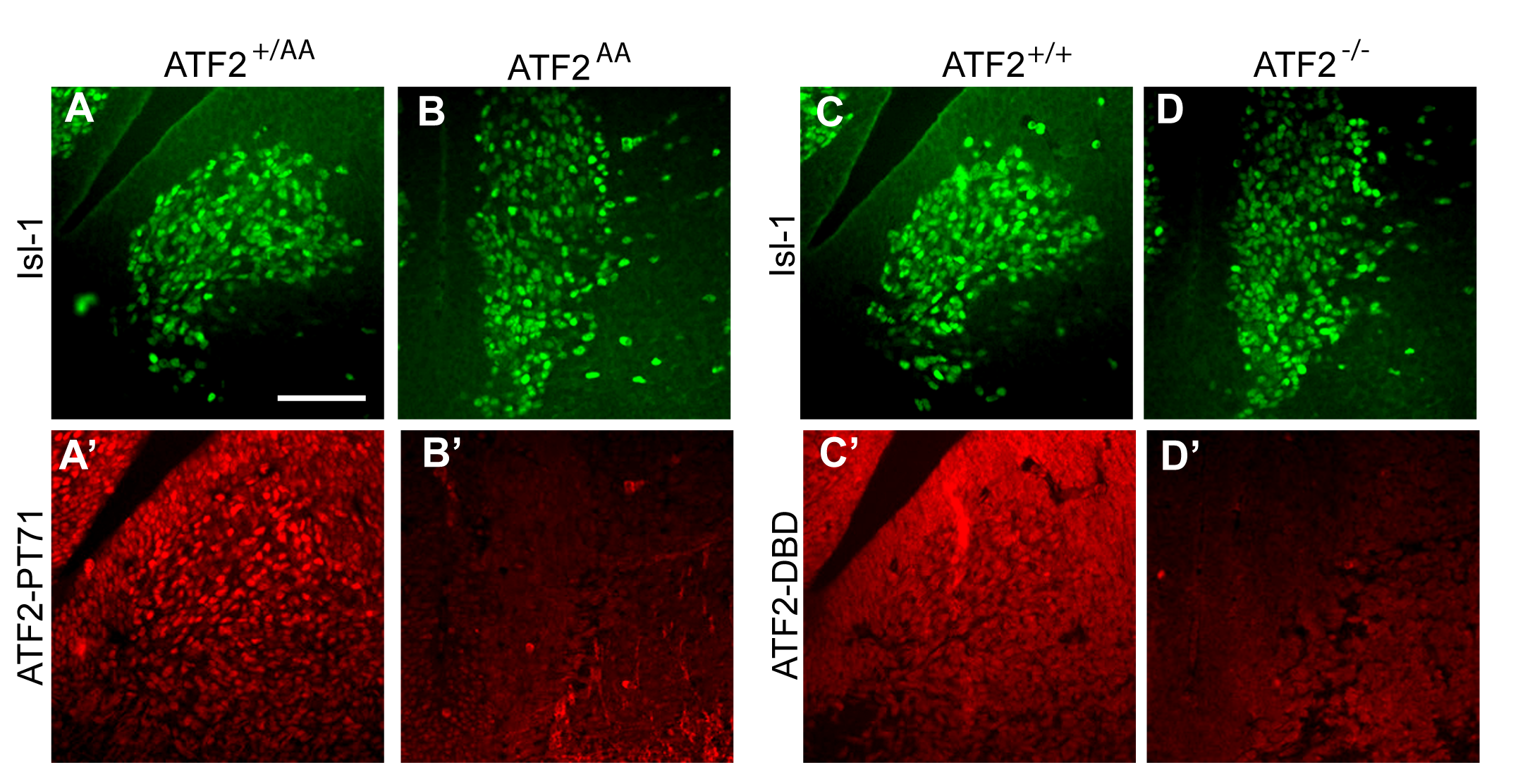

Supplement: Figure S2 — Epitope specificity of ATF2 antibodies. (A-D') E11.5 transversal brainstem sections at the level of the facial branchiomotor neurons were fluorescently stained with antibodies against Isl-1 (green, A–D) and phospho-ATF2 at Thr71 (ATF2-PT71, red, A', B') or the DNA binding domain of ATF2 (ATF2-DBD, red, C', D'). ATF2-PT71 positive signals were found in Atf2+/AA (A') but not in Atf2AA neurons (B'). ATF2-DBD positive signals were found in Atf2+/+ (C') but not in Atf2−/− neurons (D'). Bar: 100 µm. (TIF) [file pone.0019090.s002.tif]

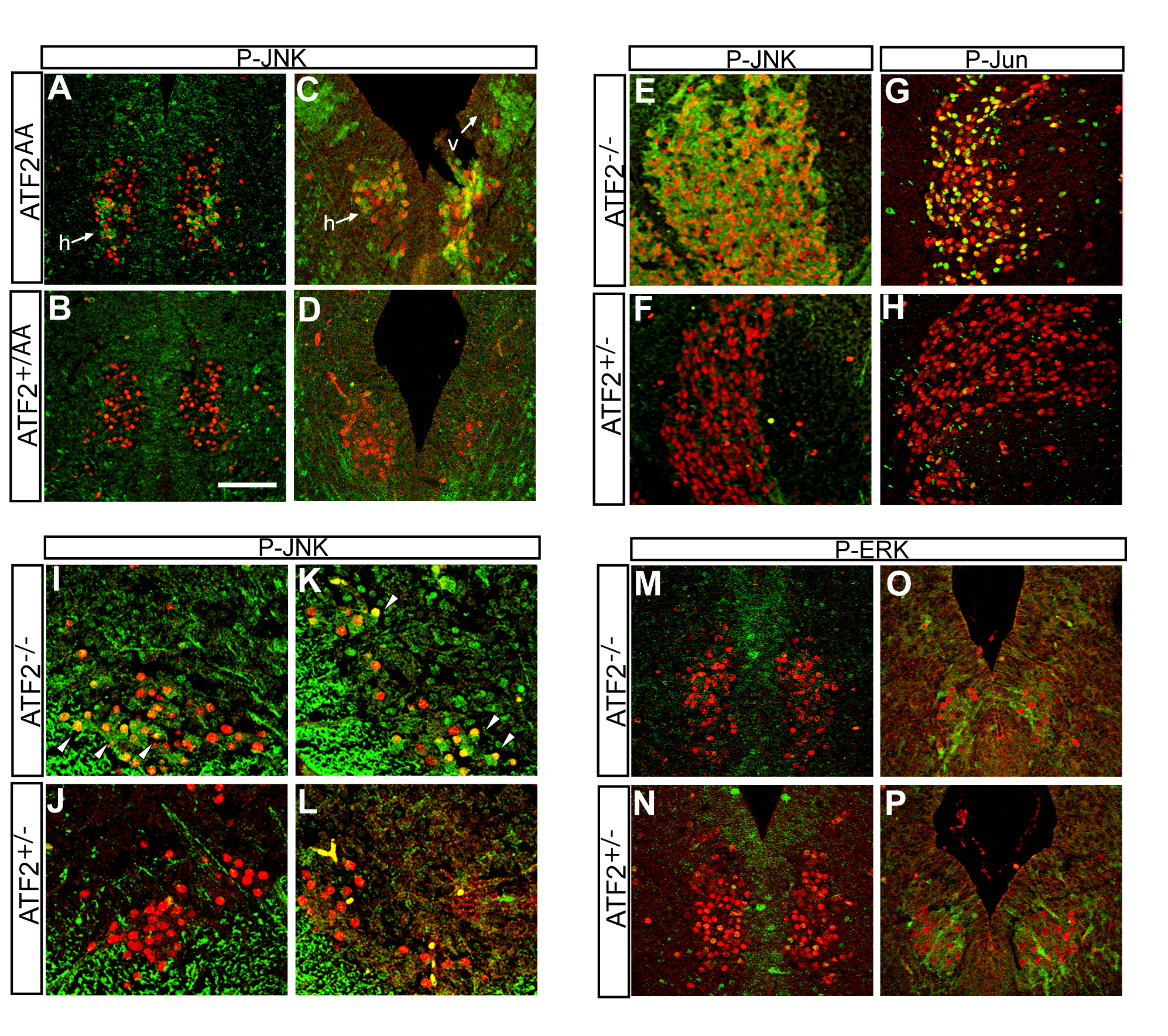

Supplement: Figure S3 — Hyperphosphorylation of JNK and c-Jun in ATF2 mutant motoneurons. (A–D) Hyperphosphorylation of JNK (green; Isl-1, red) in hypoglossal (h) and vagal (v) motoneurons in Atf2AA embryos compared to control Atf2+/AA littermate at E12.5 (A and B) and E14.5 (C and D). (E–H) Hyperphosphorylation of JNK (E and F) and c-Jun (G and H) (green; Isl-1, red) in Atf2−/− facial branchiomotor neurons at E12.5. (I–L) Hyperphosphorylation of JNK (green, Isl-1 in red) in Atf2−/− C1 motoneurons of the spinal cord (arrowheads) at E13.5 (I and J) and E15.5 (K and L). (M–P) Fluorescence immunostaining of hypoglossal motoneurons against P-ERK1/2 (Thr202/Tyr204) (green) and Isl-1 (red). No differences were observed between Atf2−/− and Atf2+/− embryos at E12.5 (M and N) or E14.5 (O, P). Bar: 100 µm. (TIF) [file pone.0019090.s003.tif]
